# Quantifying uncertainty of predictions from cancer progression models

**DOI:** 10.1093/bioinformatics/btag526

**Published:** 2026-07-24

**Authors:** Yanren Linda Hu, Simon Pfahler, Andreas Lösch, Stefan Vocht, Stefan Hansch, Kevin Rupp, Niko Beerenwinkel, Tilo Wettig, Rudolf Schill, Rainer Spang

**Affiliations:** Department for Statistical Bioinformatics, University of Regensburg, Regensburg 93053, Germany; Department of Physics, University of Regensburg, Regensburg 93040, Germany; Department for Statistical Bioinformatics, University of Regensburg, Regensburg 93053, Germany; Department for Statistical Bioinformatics, University of Regensburg, Regensburg 93053, Germany; Department for Statistical Bioinformatics, University of Regensburg, Regensburg 93053, Germany; Department of Biosystems Science and Engineering, ETH Zürich, Basel 4056, Switzerland; Department of Biosystems Science and Engineering, ETH Zürich, Basel 4056, Switzerland; Department of Physics, University of Regensburg, Regensburg 93040, Germany; Department of Biosystems Science and Engineering, ETH Zürich, Basel 4056, Switzerland; Department for Statistical Bioinformatics, University of Regensburg, Regensburg 93053, Germany

## Abstract

**Motivation:**

Cancer progresses through the accumulation of genomic events. Cancer progression models such as Mutual Hazard Networks (MHNs) describe this dynamic, enabling prediction of temporal event positions and patient-specific risks of acquiring mutations. However, current MHN analyses rely on single most likely models and do not quantify the uncertainty inherent to parameter estimation. Assessing forecast stability is essential before using them to anticipate treatment-relevant mutations, adapt targeted therapies, or prioritize monitoring of patients at elevated progression risk.

**Results:**

We address a key prerequisite for the responsible clinical use of cancer progression models by making MHN-derived predictions uncertainty-aware. We present a Bayesian framework for MHN that uses Markov Chain Monte Carlo to sample from the posterior distributions of model parameters and derived predictions. For practical use we implemented the *Random-Walk Metropolis*, *Metropolis-Adjusted Langevin Algorithm* (MALA), and *simplified manifold MALA* samplers as part of the existing mhn Python package. Only MALA and smMALA were successful in sampling from MHN posteriors, with MALA performing best. While most MHN parameters and predictions showed low posterior variance, a small subset displayed greater variability across the posterior distribution. This differentiation cannot be obtained from a single most likely model, emphasizing the need for uncertainty quantification, especially in clinical contexts. As an illustrative example, posterior sampling identified a subgroup of STK11$-$, KRAS$+$ lung adenocarcinoma patients with a high predicted short-term risk—with low variance across posterior samples—to develop an STK11 mutation. This subgroup exhibited poorer survival under immunotherapy, resembling patterns observed in STK11+ patients.

**Availability and implementation:**

Our implementation is part of version 1.2.0 of the mhn package (https://github.com/spang-lab/LearnMHN). All analyses including the code to produce all figures in this article can be found under https://github.com/huy29433/MCMC-sampling-for-MHN (https://doi.org/10.5281/zenodo.21160219).

## 1 Introduction

Cancer progresses through the accumulation of somatic genomic alterations. These alterations interact, shape tumor behavior, and influence treatment response and disease progression. Understanding how such events accumulate over time is therefore central to both cancer biology and clinical oncology.

Cancer progression models describe this process by modeling the dependencies between the genomic events that drive tumor development. These dependencies reflect both positive and negative synergies between the events. Moreover cancer progression models reveal frequently occurring mutational patterns and, in some cases, predict events that are likely to occur soon (Diaz-Colunga and Diaz-Uriarte 2021).

Direct validation of predicted future mutations, e.g. by repeat tissue sampling, is currently not feasible as repeated invasive biopsies raise ethical and clinical concerns (Mannelli 2019). Instead, predictions can be assessed indirectly by testing whether patients who are currently mutation-negative but predicted to acquire a mutation soon show similar prognosis or treatment response as patients who already carry that mutation.

From a clinical perspective, such forecasts are potentially valuable. Anticipating treatment-relevant mutations could inform therapy adaptation, risk stratification, or intensified monitoring.

State-of-the-art cancer progression models include but are not limited to, Conjunctive Bayesian Networks (Gerstung *et al.* 2009, 2011, Montazeri *et al.* 2016), Hidden Extended Suppes-Bayes Causal Networks (Angaroni *et al.* 2022), Network Aberration Models (Hjelm *et al.* 2006), Hypercubic Transition Path Sampling (Johnston and Williams 2016, Aga *et al.* 2024), Hypercubic inference of hidden Markov models (Moen and Johnston 2023), and Mutual Hazard Networks (MHNs):

MHNs model tumor progression as a continuous process in which mutations accumulate irreversibly. (Other genomic events, like copy number alterations, are also possible.) Each mutation accumulates according to a specific rate that is modulated by the presence of other mutations (Schill *et al.* 2019). Since an extension by Schill *et al.* (2024), MHNs additionally model the observation of the tumor as a random event dependent on the genomic profile. Further MHN variants describe the forming of metastases (Rupp *et al.* 2024), infer dynamics from phylogenetic trees (Luo *et al.* 2023), enable training on large datasets through approximation (Pfahler *et al.* 2026), or incorporate timing information (Chen 2023).

From a trained MHN we can infer key properties of tumor dynamics: It reconstructs tumor histories by inferring the most likely order in which mutations occurred. It can generate artificial tumor trajectories showing temporal trends. Moreover, an MHN can predict the next mutation to occur in patients, given their current genotype. Finally, to patients who do not yet have a certain mutation, it assigns the risks of developing it in the near future.

For cancer progression models to gain practical and clinical credibility, it is essential to assess the uncertainty associated with both the model parameters and the predictions derived from them. So far, only the single most likely MHN with respect to a dataset has been considered, which provides no insight into the robustness and reliability of the inferred tumor dynamics. Without this distinction, model-derived predictions cannot be used with confidence in clinical decision making.

This motivates a Bayesian perspective on the model: Here, the parameters are random variables that depend on the modeled data. Their distribution—the posterior distribution—holds uncertainty information beyond a single most likely point estimate. This uncertainty naturally propagates to uncertainty in model predictions.

Here, we analyze the full posterior distribution of MHNs and propagate parameter uncertainty to simulated tumor trajectories and patient-specific forecasts. Because MHNs’ parameter space is high-dimensional and highly correlated, efficient sampling methods are required. We therefore implement and compare Markov Chain Monte Carlo (MCMC) algorithms tailored to MHNs, including Random-Walk Metropolis (RWM), the Metropolis-Adjusted Langevin Algorithm (MALA), and simplified manifold MALA (smMALA), and identify MALA as a practical approach for posterior inference. Using posterior samples, we assess the stability of model parameters and derived predictions.

Bayesian inference and uncertainty quantification have already previously been applied to other cancer progression models: Angaroni *et al.* (2022), Johnston and Williams (2016), and Aga *et al.* (2024) leverage MCMC for model training. Moen and Johnston (2023) investigate uncertainty from data sampling using bootstrap resampling. For Hypercubic Transition Path Sampling models, posterior uncertainty has been investigated and propagated to predictions like event acquisition orderings, likely progression paths (Johnston and Williams 2016, Greenbury *et al.* 2020, Aga *et al.* 2024), and risk stratification (Johnston *et al.* 2019).

In this work, we extend uncertainty quantification via posterior analysis to MHN, which are not inherently Bayesian. We investigate how this uncertainty propagates to forecasts with potential clinical implications. Finally, we show that uninformative penalties can lead to ambiguous posteriors.

## 2 Methods and resources

We first describe Mutual Hazard Networks, originally introduced by Schill *et al.* (2019, 2024) as cancer progression models fitted via regularized maximum likelihood estimation. We then provide a brief introduction to Markov Chain Monte Carlo and the sampling algorithms used in this article. Finally, we describe how this Bayesian framework is applied to Mutual Hazard Networks and introduce the datasets used throughout the article.

### 2.1 Mutual hazard networks

Mutual Hazard Networks (MHNs) are machine-learning models that are trained on cross-sectional genomic data. Each tumor in the dataset is represented as a binary vector that encodes presence or absence of *n* irreversible cancer progression events such as point mutations in certain oncogenes.

**Figure 1:**
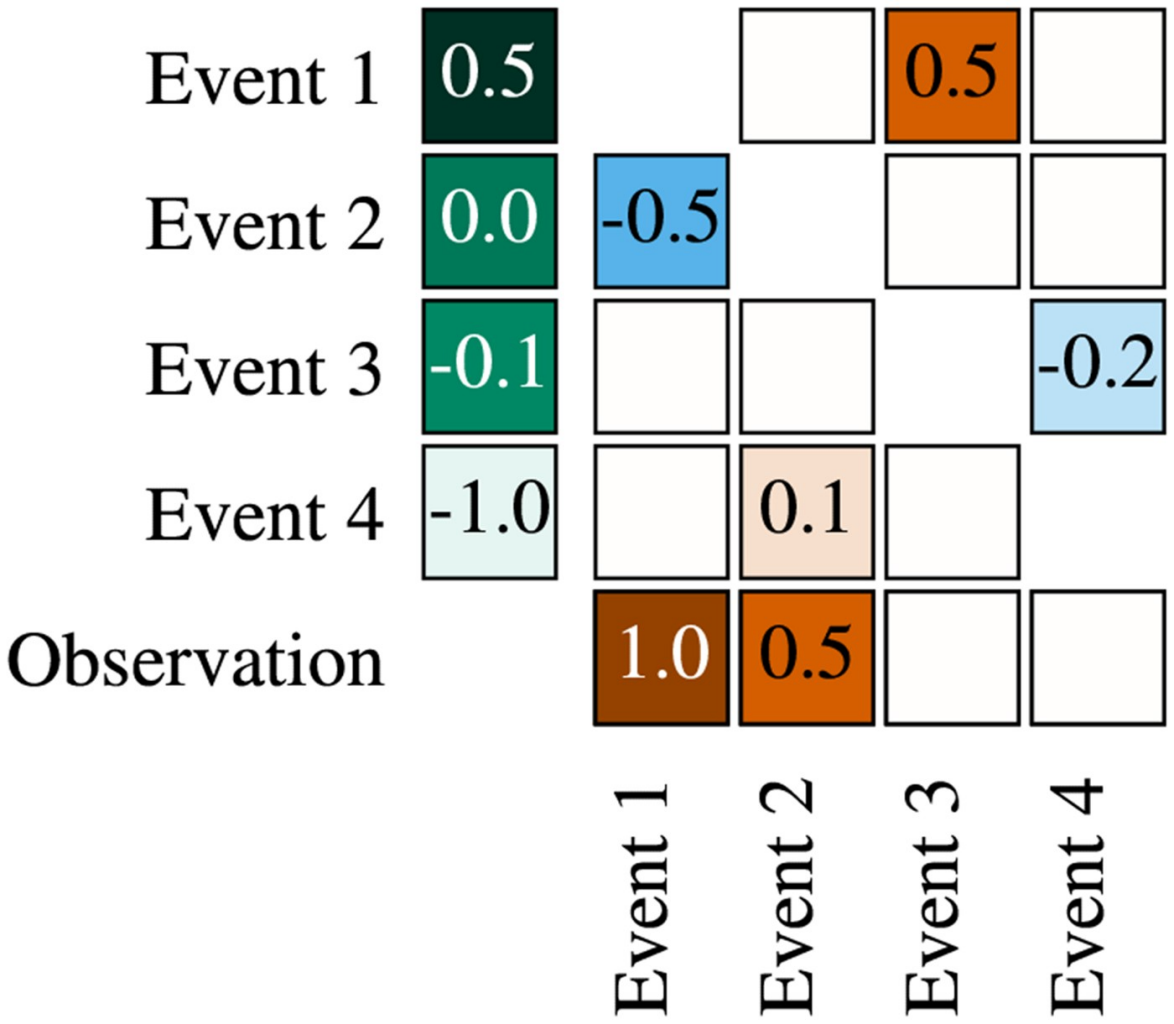
Schematic of an MHN parameter matrix for four events. Base rates $\theta_{11},\ldots \theta_{44}$ of events are plotted in the left column in green. The main matrix shows influence rates $\theta_{ij}$ between events. Orange cells visualize promoting and blue cells visualize inhibiting effects between events. For example, Event 3 promotes the accumulation of Event 1 ($\theta_{13}>0$), while Event 1 inhibits the accumulation of Event 2 ($\theta_{21}<0$). The bottom row visualizes the effect $\omega_{1},\ldots ,\omega_{4}$ of events on the clinical observation of a tumor.

MHNs were originally introduced by Schill *et al.* (2019). During training an MHN fits n×n parameters ${\left(\theta_{ij}\right)}_{i,j=1,\ldots ,n}$ (see Fig. 1) that can be interpreted as follows:

- The logarithmic *base rates* ${\left(\theta_{ii}\right)}_{i=1,\ldots ,n}$ represent the spontaneous accumulation rate of an event.
- The logarithmic *influence factors* ${\left(\theta_{ij}\right)}_{i,j=1,\ldots ,n,\;i\neq j}$ represent the influence of the presence of event *j* on the accumulation of event *i*.

Schill *et al.* (2024), extended MHN by additionally modeling the observation of a tumor, introducing an extra set of parameters ${\left(\omega_{i}\right)}_{i=1,\ldots ,n}$ (see Fig. 1):

- The logarithmic *observation effects* ${\left(\omega_{i}\right)}_{i=1,\ldots ,n}$ represent the influence of the presence of event *i* on the observation rate of the tumor.

The parameters θ are fitted using regularized maximum likelihood estimation by maximizing


(1) log p(D∣θ)+λr(θ),

with a dataset *D* and a penalty term r(θ) that is weighted by a factor λ found via cross-validation.

#### 2.1.1 Penalties

For MHN, usually the sparsity-enforcing L1 penalty


(2) $$\sum_{i\neq j}|\theta_{ij}|+\sum_{i}|\omega_{i}|,$$

or, more recently, a symmetry-favoring variant of it, the *symsparse* penalty


(3) $$\sum_{i<j}\sqrt{\theta_{ij}^{2}+\theta_{ji}^{2}-\theta_{ij}\theta_{ji}}+\sum_{i}|\omega_{i}|,$$

is used (Schill *et al.* 2019, 2024).

Here we introduce an L2 variant of (3) that additionally penalizes the base rates:


(4) $$\sum_{i<j}\left(\theta_{ij}^{2}+\theta_{ji}^{2}-\theta_{ij}\theta_{ji}\right)+\sum_{i}\theta_{ii}^{2}+\sum_{i}\omega_{i}^{2}.$$

It corresponds to a generic L2 penalty on the base rates and observation rates but favors reciprocal effects with the same strength and sign. Here, we will call it the *symL2* penalty.

The optimal regularization strength λ for MHN training is usually found through cross-validation. We will follow the strategy from Schill *et al.* (2024) and perform 5-fold cross validation on 9 logarithmically spaced λ from $1/|D|\cdot 10^{-4}$ to $1/|D|\cdot 10^{-1}$ with |D| the size of the dataset. The final λ used for training will be picked according to the one-standard error rule (Hastie *et al.* 2009).

#### 2.1.2 Chronological event positions and event risks

Based on the parameters of a fitted MHN, we can use the efficient simulation algorithm from the mhn package (Vocht *et al.* 2026), based on Gillespie’s algorithm (Gillespie 1977) to simulate artificial tumor trajectories. We can use this to analyze patterns of the tumors and make predictions about the genotypes according to the model: By simulating a large number of tumors from onset to observation, we can investigate whether specific events tend to happen earlier or later in a tumor’s development. Further, by simulating tumors starting from a patient’s genotype, we can estimate the risks of additional events happening within a certain timeframe.

These predictions may have clinical relevance and therefore require rigorous uncertainty quantification. To this end, we adopt a Bayesian perspective by viewing the inferred parameters as a random variable conditioned on the observed dataset.

From this perspective, the regularized maximum likelihood estimate of an MHN corresponds to the maximum of its posterior distribution and the penalty corresponds to a prior distribution. Using this, we can go beyond the maximum likelihood point estimate and sample from the full posterior of an MHN.

Since this posterior distribution is high-dimensional and highly correlated, we approximate it using samples drawn via Markov Chain Monte Carlo. We can then analyze the distribution of the samples and of predictions derived from them.

### 2.2 Markov Chain Monte Carlo

Markov Chain Monte Carlo (MCMC) algorithms are used to sample from (probability) distributions *p* that cannot easily be sampled from directly. They construct a discrete-time Markov chain on the sample space whose stationary distribution equals *p*.

The simplest and most commonly used MCMC algorithm, the *Random-Walk Metropolis* (RWM), transitions between states of the Markov chain in two steps: In the *proposal step*, a candidate $\theta^{\prime }$ is proposed based on the current state θ and some *proposal distribution* $q(\theta^{\prime }|\theta )$, commonly a Gaussian distribution centered around the current value. (In multi-dimensional space, one value corresponds to one parameter vector, or in our case one MHN.) Then, in the *acceptance step*, this candidate $\theta^{\prime }$ is accepted if the *acceptance ratio* $\left(p(\theta^{\prime })q(\theta |\theta^{\prime }))/(p(\theta )q(\theta^{\prime }|\theta )\right)$ is greater than a uniformly random number between 0 and 1. Otherwise the candidate is rejected and the old value is reused as the new value [see Tierney (1994)]. While this algorithm is robust and easy to implement, in high dimensions it can be very slow to converge and can produce highly correlated samples [see Roberts and Rosenthal (1998); Girolami and Calderhead (2011)].

The *Metropolis-Adjusted Langevin Algorithm* (MALA) exploits information about the distribution in order to make better proposals. It is based on discretized approximations of Langevin diffusions. The Gaussian from which the new proposed value is drawn here is not centered at the current value, but instead is shifted in the direction of the gradient of log p, scaled by a step size ϵ:


(5) $$q(\theta^{\prime }|\theta )=N\left(\theta +\frac{\epsilon }{2}\frac{\partial \;\text{log}\;p(\theta )}{\partial \theta },\epsilon I\right)$$

This will produce more informed proposals drawn from “higher probability regions,” which will then be more often accepted. In fact, Roberts and Rosenthal (2001) showed that, while an acceptance rate of around 23% of the proposals is optimal for convergence of the Random-Walk Metropolis, for MALA the optimal acceptance rate is around 57%. [While Roberts and Rosenthal (2001) only prove these results for very specific probability distributions, they suggest that they can be still be useful guidelines for more general distributions.] Therefore MALA will in general require less steps to converge to *p*, especially in higher dimensions. For MHN, the gradient can efficiently be calculated in one step together with the likelihood. In fact, in the current implementation (Vocht *et al.* 2026), this happens by default, which is why upgrading from Random-Walk Metropolis to MALA does not add significant computational complexity. See Algorithm SA1, available as supplementary data at *Bioinformatics* online for pseudocode of MALA applied to MHN.

*Manifold MALA* (mMALA), introduced by Girolami and Calderhead (2011), extends MALA by a proposal that adapts to some geometry at the current value. Distances for the proposal function are not measured via the usual Euclidean metric, but via a Riemannian metric which is induced by some Riemannian metric tensor G(θ). In its simplified version (smMALA), that assumes constant curvature, the proposal function becomes


(6) $$q(\theta^{\prime }|\theta )=N\left(\theta +\frac{\epsilon }{2}G{\left(\theta \right)}^{-1}\frac{\partial \;\text{log}\;p(\theta )}{\partial \theta },\epsilon G{\left(\theta \right)}^{-1}\right).$$

As a metric tensor for sampling from a posterior distribution p(·)∼p(D∣·)pr(·) with prior pr (as its Bayesian counterpart, the prior is directly induced by the penalty), Girolami and Calderhead (2011) suggest the Fisher information matrix I(θ) of p(D∣·) minus the Hessian of the log-prior:


(7) G(θ)=I(θ)−H(logpr(θ))

with


(8) $$I{\left(\theta \right)}_{ij}=E\left[\frac{\partial \;\text{log}\;p}{\partial \theta_{i}}\frac{\partial \;\text{log}\;p}{\partial \theta_{j}}|\theta \right].$$

The reason for this choice is that the Fisher information matrix (FIM) encodes the distance of two densities on the statistical manifold of parametrized probability density functions. In simpler terms, instead of considering two values similar if their parameters are close to each other, under the FIM as a metric tensor they are considered similar, if they induce similar probability densities. The induced metric is then also independent from reparameterization. In order to also capture prior informativeness in the metric tensor, the negative Hessian of the log-prior is added. See Algorithm A2, available as supplementary data at *Bioinformatics* online for pseudocode of smMALA applied to MHN.

However, there are some disadvantages to (s)mMALA: For each step, the FIM has to be computed. Even though this can be done analytically for MHNs (see Supplementary 1.1, available as supplementary data at *Bioinformatics* online), this introduces significant overhead, possibly rendering sampling impossible for big models. Also, it can only be applied with somewhat “well-behaved” priors for which G(θ) is positive definite, excluding the L1 and symsparse priors induced by Equations (2) and (3). For a detailed explanation of this see Supplementary 1.3, available as supplementary data at *Bioinformatics* online.

#### 2.2.1 MCMC diagnostics

In MCMC algorithms, the sampled distribution converges to the desired distribution as the number of steps tends to infinity. In practice, however, one wants to have an estimate of how many steps are needed to get close enough to this distribution. One diagnostic of this is the potential scale reduction factor $\hat{R}$ (Gelman and Rubin 1992). It is based on starting multiple chains from different initializations and comparing the variance of the samples of all chains with the variance of the samples within the chains. It is always >1 and suggests better convergence the closer it is to 1. Vehtari *et al.* (2021) improved it to detect bad convergence for more general distributions and samplers. They recommend to run the sampling until $\hat{R}<1.01$.

Within one MCMC chain, samples are usually autocorrelated. Roughly speaking, the *effective sample size* (ESS) describes this autocorrelation by estimating how many *uncorrelated* draws from the distribution would contain the same amount of information.

For both $\hat{R}$ and the ESS we use the arviz Python package by Kumar *et al.* (2019). (We use the default method for both functions, which for arviz.rhat is "rank" and for arviz.ess is "bulk".)

### 2.3 MCMC sampling with the mhn.mcmc module

We implemented the three MCMC algorithms RWM, MALA, and smMALA as a submodule of the mhn package (Vocht *et al.* 2026). Penalties, like the symsparse or the L1 penalty, whose strengths are found in cross-validation, are translated into the corresponding Bayesian priors. (Tuning λ in cross-validation is standard in machine learning, but not in a classical Bayesian framework, where λ would be treated as a hyperparameter with its own prior. We chose to keep this optimization-based calibration to ensure that MCMC quantifies uncertainty for MHN models as originally defined.) The implementation includes automatic tuning of the step size and automatic halting as soon as, e.g. the desired $\hat{R}$ is reached.

While other RWM and MALA implementations exist (e.g. Abadi *et al.* 2015, Clerx *et al.* 2019), our implementation also includes smMALA, which to our knowledge does not exist as open source code so far. Moreover, our implementation is tailored to MHN and uses its joint likelihood- and gradient evaluation and therefore enables faster sampling than out-of-the-box solutions can. See Supplementary S3, available as supplementary data at *Bioinformatics* online for a benchmark analysis of the scaling of one MALA step. Finally, this integration allows users to benefit from MCMC sampling for MHN without requiring in-depth knowledge of the underlying mathematics or implementation details.

In this article, we sampled 10 chains for each trained MHN. To reduce memory usage and the cost of downstream predictions, we applied *thinning*, i.e. storing only every 100th sample. (We found the resulting ESS to be sufficiently large, see Table 1.) We sampled until either a maximal $\hat{R}$ of <1.01, or a total of 1 000 000 steps was reached. The initial values for the Markov chains were drawn from either a Laplace distribution (for models trained with the L1 or symsparse penalty) or a Gaussian distribution (for models trained with the symL2 penalty), approximating the priors induced by the penalties. For all diagnostics and analyses we discarded the first 20% of the steps as burn-in. (We chose this as a starting heuristic and saw that it produced sufficiently small $\hat{R}$, see Table 1).

**Table 1:** Effective sample size (ESS) and $\hat{R}$ diagnostic per parameter and number of steps for the MCMC sampling runs from the posteriors of two MHNs trained on the LUAD and COAD dataset, respectively.

Dataset	**ESS** median [**min,** max]	$\hat{R}$ median [min, **max**]	Steps (without burn-in)
LUAD	5702.39 [**1190.78**, 10873.37]	1.00106 [0.99971, **1.00970**]	146 400
COAD	4359.84 [**1034.83**, 9662.62]	1.00134 [0.99992, **1.00997**]	108 800

The bold values show the minimum for ESS and the maximum for R hat.

### 2.4 Datasets

The results we show in this article are based on MHNs that were trained on the same datasets as in Schill *et al.* (2024): Somatic mutation data of 3662 lung adenocarcinoma (LUAD) and of 2269 colon adenocarcinoma (COAD), respectively. These datasets were collected by the Memorial Sloan Kettering Cancer Center (Nguyen *et al.* 2022) and retrieved through AACR GENIE (Sweeney *et al.* 2017). We selected the 12 most commonly affected genes and followed Schill *et al.* (2024)’s preprocessing steps.

## 3 Results

### 3.1 MHN posterior sampling enabled by gradient-based MCMC

In order to attempt posterior sampling with Markov Chain Monte Carlo, we applied the three sampling algorithms RWM, MALA, and smMALA to an MHN trained on the LUAD dataset with the symL2 penalty given by Equation (4).

We chose this penalty because, as noted above, smMALA requires a sufficiently “well-behaved” prior in our setting. This excludes priors induced by penalties that do not penalize all parameters or that involve L1 regularization. Consequently, unlike the other two samplers, smMALA cannot be used in combination with the standard MHN penalties given by Equations (2) and (3).

**Figure 2:**
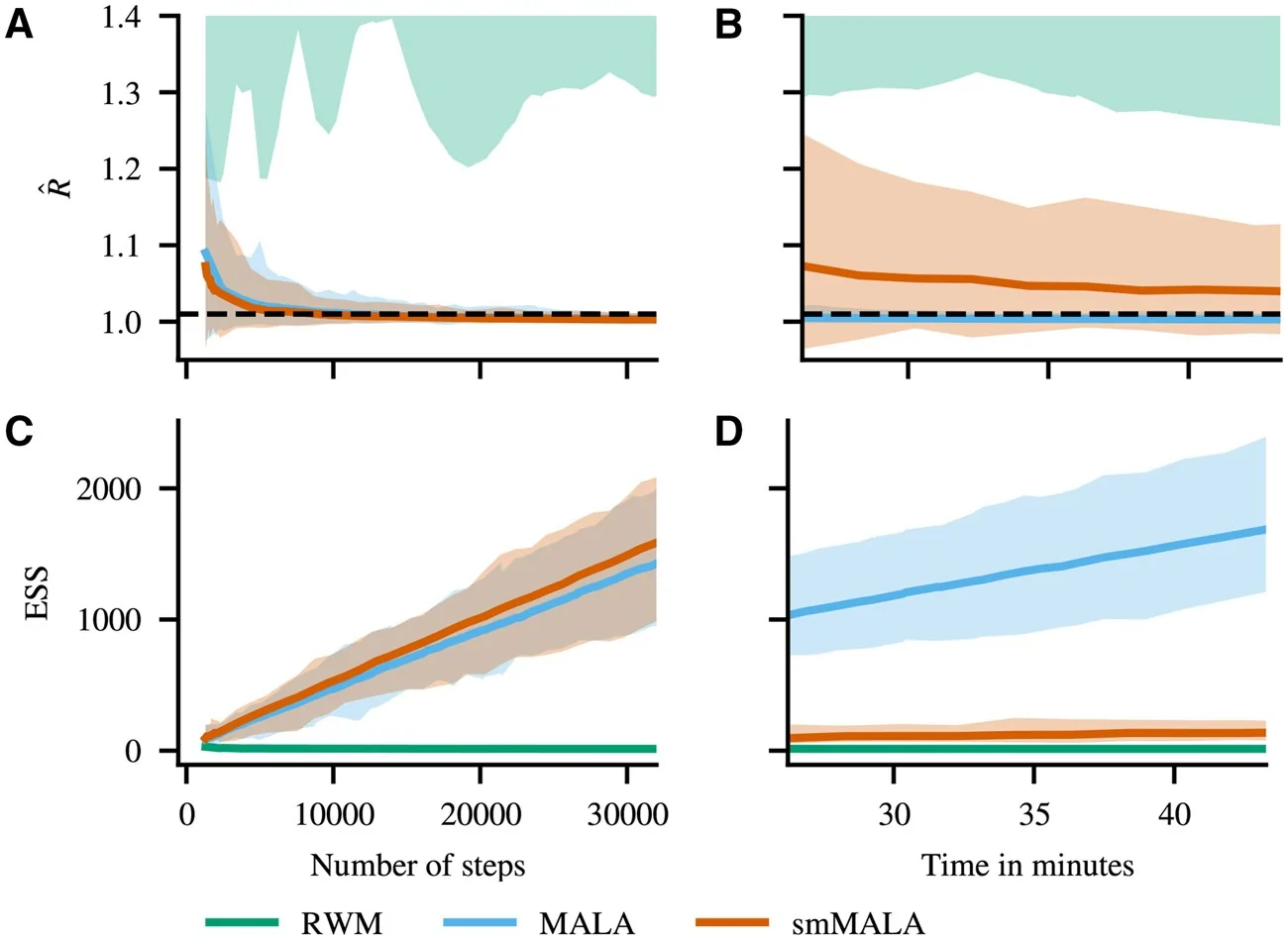
Mean, minimum, and maximum $\hat{R}$ and effective sample size (ESS) of all parameters from MCMC samplers applied to an MHN trained on the LUAD dataset with the symL2 penalty [Equation (4)]. The metrics for RWM, MALA and smMALA are plotted against number of steps and time. Diagnostics were evaluated after discarding the first 20% of the steps as burn-in.

Even after 1 000 000 steps, RWM failed to reach satisfactory convergence (Fig. 2A). Its maximal $\hat{R}$ remained at 2.6047, showing that the chains did not mix sufficiently to approximate the posterior distribution.

In contrast, we were able to achieve MCMC posterior sampling with the two gradient-based samplers, MALA and smMALA. Both reached satisfactory convergence within a few tens of thousands of steps: MALA reached a maximal $\hat{R}$ of <1.01 after 38 000 steps, and smMALA after only 32 000 steps.

Samples from the gradient-based samplers were also considerably less autocorrelated (Fig. 2C): Even after 1 000 000 steps, the minimal ESS for RWM was only 11.86. In contrast, after 32 000 steps, MALA achieved a minimal ESS of 953.49 and smMALA a minimal ESS of 987.21.

However, in the case of smMALA, this performance came at a substantially increased computational cost: A single RWM or MALA step took 0.0676*s* and 0.0684*s*, respectively, whereas a single smMALA took 1.21*s*, as in our case the FIM has to be computed for each step.

In the following analysis of MHN posteriors, we exclusively use MALA.

### 3.2 Parameter uncertainty

We analyzed the posteriors of MHNs trained on the LUAD and the COAD dataset with the symsparse penalty, as discussed in Schill *et al.* (2024).

In Table 1, we report the convergence and autocorrelation diagnostics of the runs and the number of steps to achieve them.

**Figure 3:**
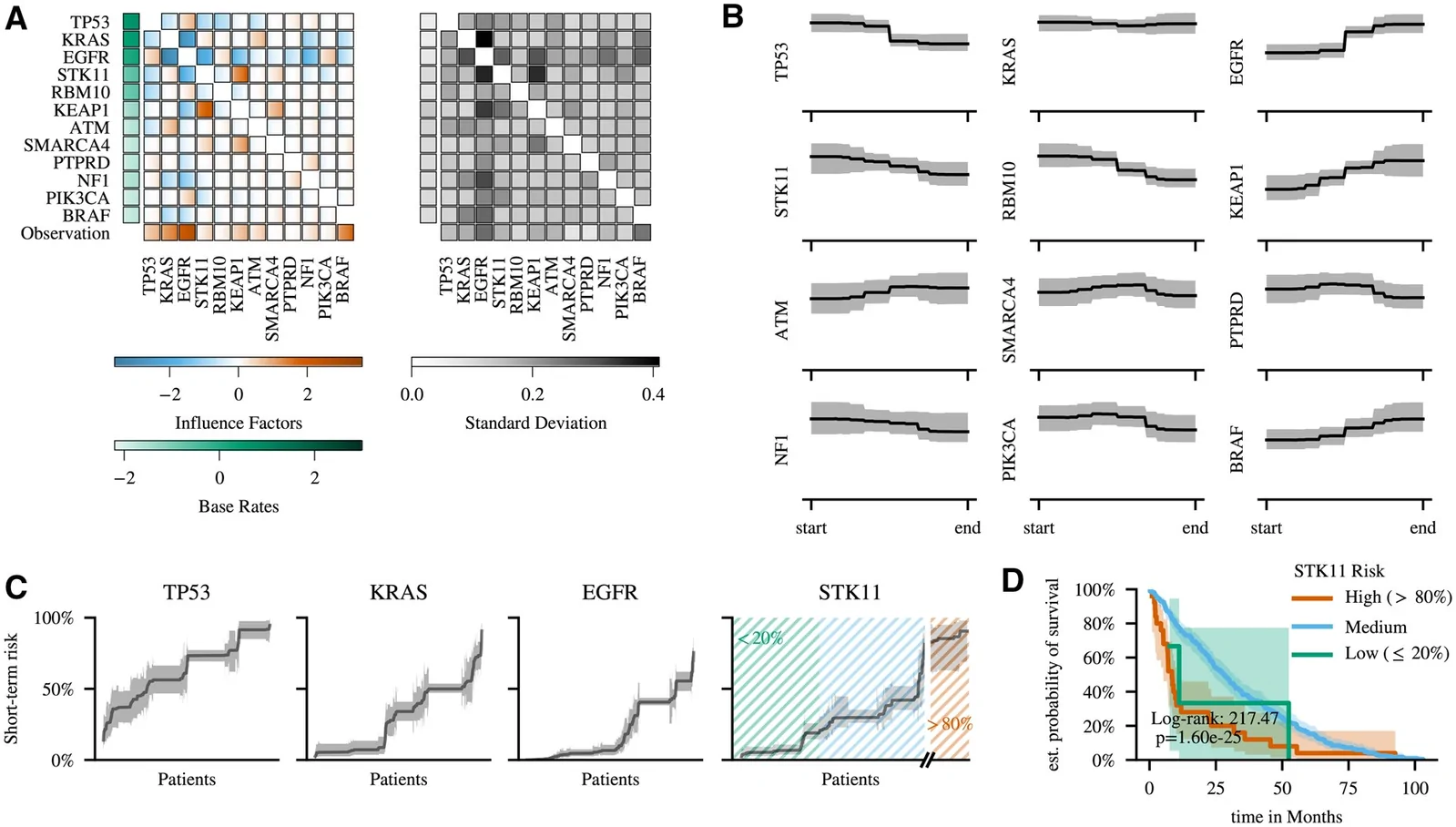
(A) Distribution and standard deviation of the parameter matrix for MHNs trained on the LUAD dataset. Each cell in the left plot shows the 90% posterior interval of the corresponding parameter as a color gradient. For example, the leftmost color in a cell corresponds to the 5% quantile of the parameter’s distribution, the middle color to its 50% quantile, the rightmost color to its 95% quantile. Influence factors and observation rates are shown in blue/orange and base rates are shown in green. Each cell in the right plot shows the standard deviation of the corresponding parameter. (B) Posterior distributions of relative event accumulation times (*x*-axis), shown as median and 90% posterior intervals. For every 100th MCMC sample, 50 000 artificial tumor trajectories were simulated, and the relative accumulation time of each event was recorded. This yields, for each event and every 100th MCMC sample, a distribution describing when the event tends to occur. The figure summarizes the posterior distribution of these event-specific accumulation-time curves. (C) Predicted short-term mutation risks (median and 90% posterior interval) across patients of the LUAD dataset, predicted by the MCMC samples (*x*-axis broken and rescaled for high-risk patients). For every 100th MCMC sample, 10 000 artificial tumors were sampled for 1 time unit in Markov time to infer a mutation risk. Only patients who do not exhibit this mutation at the time of sequencing are shown and patients are ordered by median risk. (D) Kaplan–Meier estimate of the survival of immuno-treated, KRAS-positive patients from the LUAD dataset. Survival estimates are stratified by median STK11 risk across MCMC samples.

**Figure 4:**
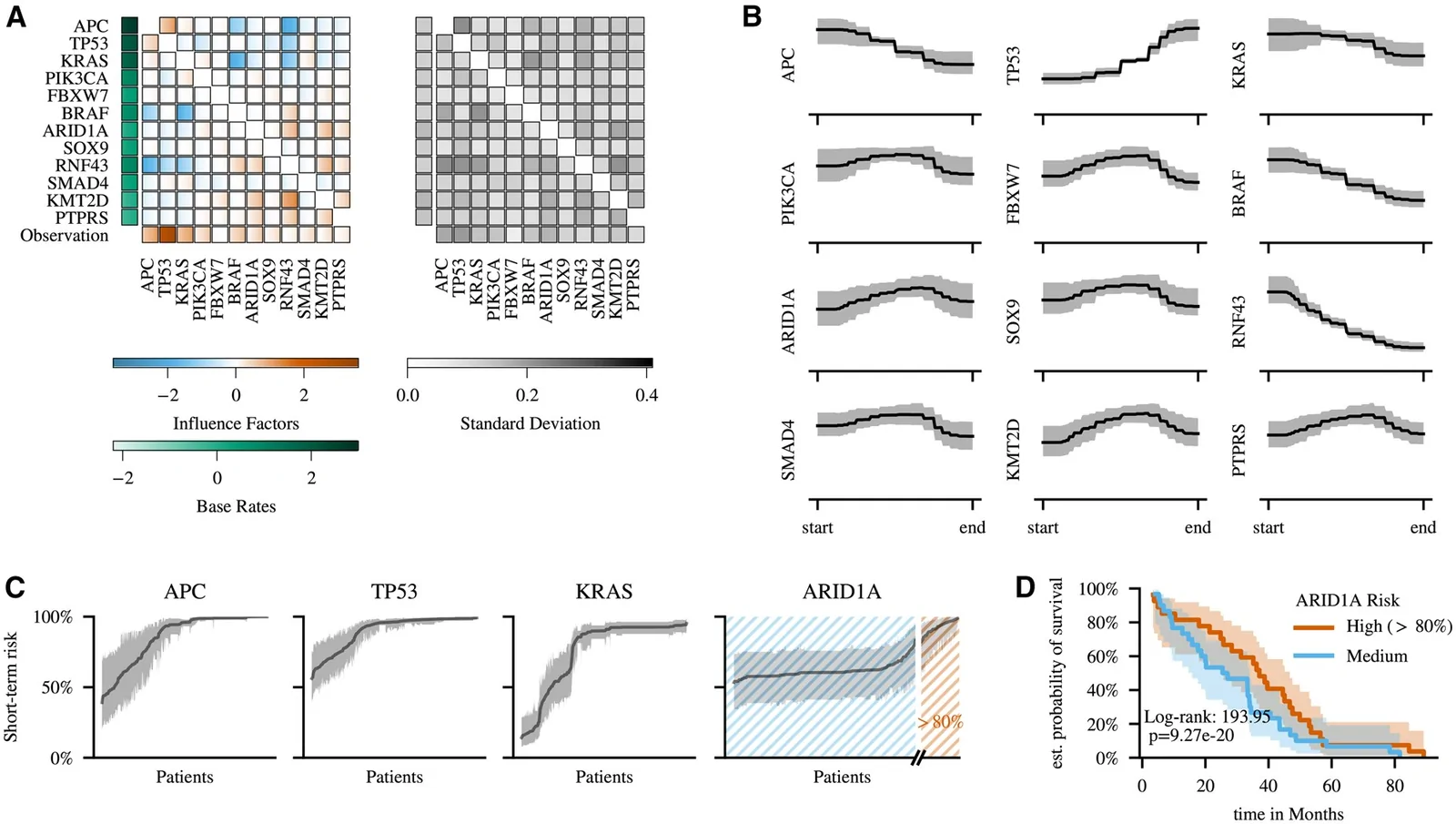
(A) Distribution and standard deviation of the parameter matrix for MHNs trained on the COAD dataset. Each cell in the left plot shows the 90% posterior interval of the corresponding parameter as a color gradient. For example, the leftmost color in a cell corresponds to the 5% quantile of the parameter’s distribution, the middle color to its 50% quantile, the rightmost color to its 95% quantile. Influence factors and observation rates are shown in blue/orange and base rates are shown in green. Each cell in the right plot shows the standard deviation of the corresponding parameter. (B) Posterior distributions of relative event accumulation times (*x*-axis), shown as median and 90% posterior intervals. For every 100th MCMC sample, 50 000 artificial tumor trajectories were simulated, and the relative accumulation time of each event was recorded. This yields, for each event and every 100th MCMC sample, a distribution describing when the event tends to occur. The figure summarizes the posterior distribution of these event-specific accumulation-time curves. (C) Predicted short-term mutation risks (median and 90% posterior interval) across patients of the COAD dataset, predicted by the MCMC samples (*x*-axis broken and rescaled for high-risk patients). For every 100th MCMC sample, 10 000 artificial tumors were sampled for 1 time unit in Markov time to infer a mutation risk. Only patients who do not exhibit this mutation at the time of sequencing are shown and patients are ordered by median risk. (D) Kaplan–Meier estimate of the survival of immunotherapy-treated patients from the COAD dataset. Survival estimates are stratified by median ARID1A risk across MCMC samples.

For both datasets, posterior standard deviation of the parameters was very low, especially in the base rates (Figs 3A and 4A). The standard deviation of influence factors and observation rates was higher when their posterior distributions were farther from zero. Parameters with comparatively high standard deviation in the lung cancer model were primarily influences that connected the EGFR mutation to other events or the observation. In the colon cancer model, posterior standard deviations were uniformly small across parameters and no mutation exhibited comparable variability.

### 3.3 Temporal event positions across posterior samples

We derived the relative temporal positions of events according to the posterior samples. To do so, we simulated 50 000 artificial tumor trajectories for every 100th MCMC sample and recorded the relative accumulation time of each event along the tumor development timeline. For each event and every 100th MCMC sample we thus obtained a distribution over the tumor development, describing when the event tends to occur.

In the posterior of the LUAD model, TP53 was consistently placed early, while EGFR was placed late (Fig. 3B). In the posterior of the COAD model, APC was placed early, KRAS a bit later and TP53 rather late (Fig. 4B). Event position distributions showed little variation, with narrow posterior intervals for these mutations.

### 3.4 Predicted high-risk and mutation-positive patients with similar prognosis

For each mutation and each patient who did not yet exhibit this mutation at sequencing time, we used the posterior samples to predict the short-term risk of mutation occurrence. To this end, for every 100th MCMC sample and each patient, we simulated 10 000 times from the patient’s genotype forward over a horizon of 1 time unit in Markov time. We recorded the proportion of simulations in which an event occurred as the event’s mutation risk.

Figures 3C and 4C show the median risks for a subset of the mutations and their 90% posterior intervals predicted from the MCMC samples for all LUAD and COAD patients, respectively.

For many mutations in both datasets, the predicted risks ranged from almost 0% to almost 100% across patients, reflecting the heterogeneity in individual risk predictions.

For the lung cancer model, TP53 and KRAS risk predictions showed wide posterior intervals for patients with an intermediate median predicted risk, with posterior intervals spanning up to almost 50 percentage points. In contrast, high- and low-risk predictions exhibited narrower intervals (<34 and <24 percentage points, respectively). For STK11, both low- and high-risk predictions were comparatively stable, with high-risk posterior intervals spanning <35 percentage points and never dropping below 60%.

We stratified KRAS-mutated, STK11-negative lung cancer patients who received immunotherapy into risk groups according to their posterior median predicted STK11 risk. Figure 3D shows the corresponding Kaplan–Meier survival estimates. High-risk patients exhibited poorer survival compared to medium-risk patients. This observation is in line with STK11-*positive* patients, that also exhibit poorer survival under immunotherapy (Skoulidis *et al.* 2018, Ricciuti *et al.* 2022), suggesting that STK11-positive and STK11-negative high-risk patients form a clinically homogenous group.

In the COAD model, APC and TP53 risk predictions showed wide posterior intervals for patients with an intermediate median predicted risk (posterior intervals spanning up to >55 percentage points), while for very high predicted risks (median >97%), the posterior interval shrunk to <9 percentage points. For ARID1A, medium-risk patients again showed notable variance in their predicted risk (posterior interval up to almost 46 percentage points), whereas high-risk predictions were comparatively stable with posterior intervals smaller than 40 percentage points and shrinking to <9 percentage points for patients with median risk above 97%.

We stratified ARID1A-negative colon cancer patients who had received immunotherapy according to their median predicted posterior risk. Figure 4D shows their Kaplan–Meier survival estimates. Patients with high predicted AIRD1A risk exhibited better survival compared to the medium-risk group. Again, this was comparable to the prognosis of ARID1A-*positive* patients (Jiang *et al.* 2020, Okamura *et al.* 2020, Tokunaga *et al.* 2020).

### 3.5 Multimodal posterior caused by uninformative penalties

**Figure 5:**
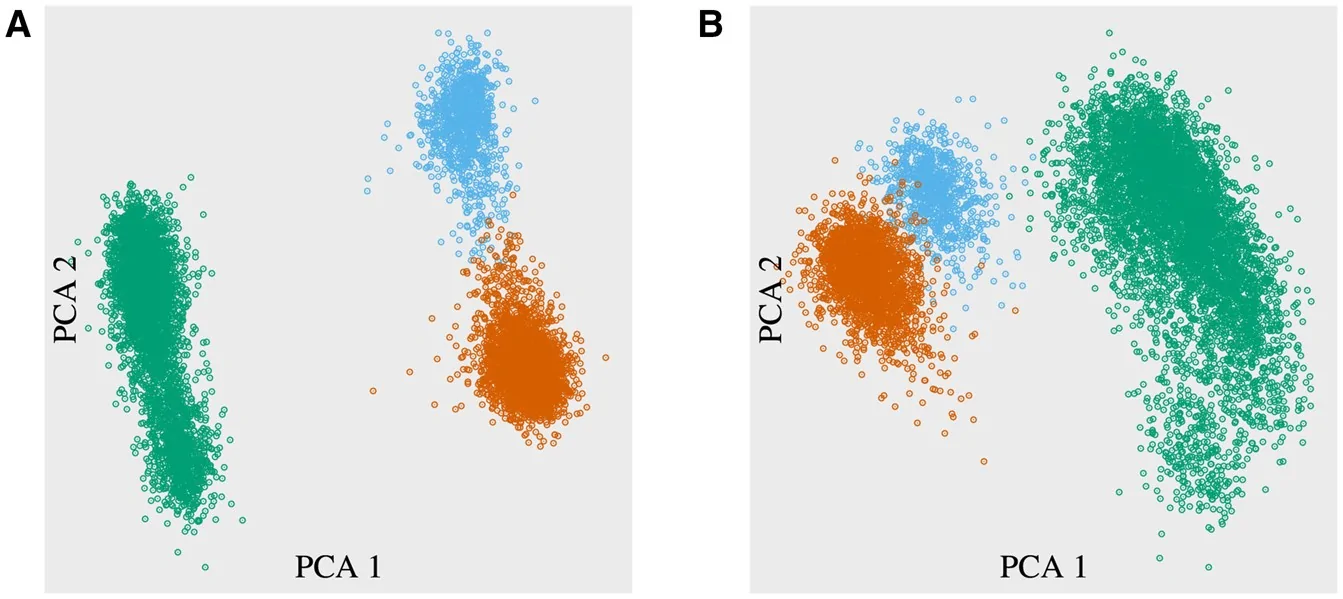
First two principal components of posterior samples from MHNs trained on the (A) LUAD and (B) COAD dataset with a non-symmetrizing L1 penalty. Every 10th sample is shown and colored by cluster. Cluster identities were assigned by applying HDBSCAN to a 2D UMAPping.

Models trained with less informative penalties, that do not promote symmetry, can exhibit ambiguous posteriors:

We sampled from the posteriors of MHNs trained on the LUAD and the COAD dataset with the L1 penalty given by Equation (2). Even after 1 000 000 steps, we could not achieve satisfactory convergence for either of those models—in contrast to the models trained with the symsparse or symL2 penalty. Analyzing the distribution of the produced samples suggests the presence of multiple modes in the models’ posterior distributions. Chains often got stuck in one of these, preventing proper exploration of the probability distribution. Some chains in fact stayed confined within one of the modes for the entire sampling process. In Fig. 5, we show the first two principal components of all MCMC samples, where the modes are clearly visible.

The modes in the LUAD L1 model differed primarily in how they resolved the co-occurrences of STK11 with KEAP1 and KEAP1 with SMARCA4: Two modes explained this with a positive influence of KEAP1 on STK11, while the third reversed this influence. Similar behavior was observed for KEAP1 and SMARC4.

In the same way, the modes in the COAD L1 model reversed directions of KMT2D and RNF43 or ARID1A and KMT2D. See Supplementary S6, available as supplementary data at *Bioinformatics* online for a detailed description of the modes.

However, many dynamics in cancer are due to positive or negative epistatic (i.e. synergistic) effects (El Tekle *et al.* 2021, Iranzo *et al.* 2022) and might instead best be explained symmetrically.

## 4 Discussion

In this work, we enabled posterior analysis of Mutual Hazard Networks (MHNs) and their predictions through the use of gradient-based MCMC samplers. For this the Metropolis-Adjusted Langevin Algorithm represented the best balance of geometrically informed but computationally efficient steps.

Posterior analysis for models trained on different datasets revealed differences in the stability of MHN-based predictions with respect to parameter estimation.

In the LUAD and COAD model, base rates showed low posterior standard deviation, whereas influence factors between mutations and on the observation exhibited comparably higher variability. Many influence factors from or to the EGFR mutation in lung cancers displayed elevated posterior standard deviation, indicating a slight uncertainty in the role of this mutation. Despite this variability in EGFR-parameters, the derived temporal positions and mutation risks remained stable. This indicates that parameter uncertainties do not necessarily propagate to model predictions.

The temporal event positions remained stable across the posterior in both datasets. They were in line with the orderings reported by Schill *et al.* (2024) and, in the case of COAD, the sequence APC → KRAS → TP53 aligned with the established adenoma-to-carcinoma progression model by Vogelstein *et al.* (2013).

In both dataset, posterior sampling identified subgroups that exhibited a stable risk to develop a certain mutation and that showed similar survival patterns as mutation-positive patients. This suggests that a high predicted mutation risk may anticipate the disease progression and potentially inform early treatment adjustments.

For models trained with less informative penalties, posterior sampling revealed multiple modes in the posterior distribution. These were due to ambiguous directions in influences between two events, where symmetric effects (as encouraged by the symsparse penalty) might be more plausible.

While posterior sampling allows us to identify predictions that are stable with respect to parameter estimation, this is not the only source of uncertainty: Model and penalty choices, as well as feature selection, introduce additional uncertainty. Even earlier, cohort selection, mutation calling, filtering, and data imputation can affect the estimated parameters and resulting predictions. For a comprehensive assessment of uncertainty, all of these steps need to be considered.

Taken together, these observations emphasize the importance of cautious interpretation of cancer progression model predictions. While they are not yet ready for clinical practice, they provide a structured framework for anticipating tumor evolution. Our results highlight the benefits of incorporating uncertainty-awareness into cancer progression modeling. In particular, differentiating between stable and unstable predictions represents a crucial step toward responsible interpretation and, eventually, practical application in clinical contexts.

## Supplementary Material

btag526_Supplementary_Data

## Data Availability

The data underlying this article are available at https://github.com/huy29433/MCMC-sampling-for-MHN.
