# Supplementary material for: Quantifying uncertainty of predictions from cancer progression models
Source: Bioinformatics. 2026 Jul 24;42(8):btag526. doi: 10.1093/bioinformatics/btag526 (PMC13437017; doi:10.1093/bioinformatics/btag526)
Supplement: Supplementary file 1 [file btag526_supplementary_data.pdf]

Supplementary Information to Quantifying Uncertainty of Predictions from Cancer Progression Models

July 14, 2026

Contents

S1 Metric Tensor for smMALA **2**
S1.1 Computing and Using the FIM for MHN 2
S1.1.1 The FIM for oMHN 2
S1.1.2 Using the Metric Tensor 3
S1.2 Approximating the FIM 3
S1.3 Positive Definiteness of the Metric Tensor 3
S1.3.1 Non-Positive Definiteness of the FIM for MHN 3
S1.3.2 (Non-) Negative Definiteness of the Prior 4

S2 Pseudocode (sm)MALA **5**

S3 MALA Scaling **6**

S4 Posterior Predictive Checks **7**
S4.1 LUAD 9
S4.2 COAD 12

S5 Mutation Risks **14**

S6 Multimodal Posterior Distributions for the L1 MHNs **16**
S6.1 LUAD 16
S6.2 COAD 18

S1 Metric Tensor for smMALA

As suggested by Girolami and Calderhead (2011), we use the Fisher information matrix minus the Hessian of the log-prior, eq. (7), as a metric tensor for smMALA.

S1.1 Computing and Using the FIM for MHN

For the computation of the Fisher information matrix, we built upon Vocht (2022, `https://github.com/StefanDevAccount/bachelorthesis`)'s implementation of the Fisher information matrix for cMHN. We improved the runtime, added a GPU implementation and generalized it to oMHN. The implementations can be found in *mhn/full_state_space/cuda_full_state_space.cu* and *mhn/full_state_space/fisher.pyx* in the `mhn` repository in version 1.2.0.

S1.1.1 The FIM for oMHN

The FIM for oMHN can directly be computed from the FIM of its corresponding cMHN: For every oMHN, there is a cMHN that describes the same probability distribution: The function

$$\begin{aligned} c : \mathbb{R}^{(n+1)\times n} &\to \mathbb{R}^{n\times n} \\ \begin{pmatrix} (\theta_{ij})_{ij} \\ (\omega_j)_j \end{pmatrix} &\mapsto \begin{pmatrix} \theta_{ij} - \omega_j \text{ if } i \neq j \\ \theta_{ii} \text{ else} \end{pmatrix}_{ij} \end{aligned} \tag{s1}$$

maps an oMHN to its corresponding cMHN. Let $p^o(D \mid \cdot)$ and $p^c(D \mid \cdot)$ describe the oMHN and cMHN likelihood function, respectively, given some dataset D. Then $p^o(D \mid \cdot) = p^c(D \mid c(\cdot))$ and

$$\frac{\partial p^o(D \mid \cdot)}{\partial \theta_{ij}} = \frac{\partial p^c(D \mid \cdot)}{\partial \theta_{ij}} \circ c \qquad \text{and} \tag{s2}$$

$$\frac{\partial p^o(D \mid \cdot)}{\partial \omega_j} = -\sum_{i\neq j} \frac{\partial p^c(D \mid \cdot)}{\partial \theta_{ij}} \circ c\,. \tag{s3}$$

Now, if $\mathcal{I}^o(\cdot)$ and $\mathcal{I}^c(\cdot)$ are the Fisher information matrices for oMHN and cMHN, respectively, we have

$$(\mathcal{I}^o(\cdot))_{\theta_{ij}\theta_{kl}} = (\mathcal{I}^c(c(\cdot)))_{\theta_{ij}\theta_{kl}} \tag{s4}$$

$$(\mathcal{I}^o(\cdot))_{\omega_j\theta_{kl}} = -\sum_{i\neq j} (\mathcal{I}^c(c(\cdot)))_{\theta_{ij}\theta_{kl}} \qquad \text{and} \tag{s5}$$

$$(\mathcal{I}^o(\cdot))_{\omega_j\omega_l} = \sum_{i\neq j}\sum_{k\neq l} (\mathcal{I}^c(c(\cdot)))_{\theta_{ij}\theta_{kl}}\,. \tag{s6}$$

S1.1.2 Using the Metric Tensor

For smMALA, we need to use the metric tensor $G(\theta) = \mathcal{I}(\theta) - H(\log \mathrm{pr})$, with $\mathcal{I}(\theta)$ the Fisher information matrix of an MHN and $H(\log \mathrm{pr})$ the Hessian of the log-prior, in multiple ways:

$G(\theta)^{-1}$ As it is positive definite, the matrix $G(\theta)$ allows for a Cholesky decomposition $G(\theta) = LL^T$ with L a lower triangular matrix. Therefore we can efficiently compute $G(\theta)^{-1}x = L^{-T}L^{-1}x$ through substitution.

$\det G(\theta)$ Through its Cholesky decomposition we also get $G(\theta)$'s determinant efficiently as the squared product of L's diagonal entries.

drawing from $N(\mu, \epsilon G(\theta)^{-1})$ This can be achieved by drawing a multidimensional standard normal vector z and transforming it with $\sqrt{\epsilon}L^{-T}z+\mu$.

S1.2 Approximating the FIM

The Fisher information matrix can be approximated by drawing a sufficiently large set of samples $S \sim p(\cdot \mid \theta)$:

$$\mathcal{I}(\theta)_{ij} = E\left[\frac{\partial \log p}{\partial \theta_i}\frac{\partial \log p}{\partial \theta_j} \,\middle|\, \theta\right] \tag{s7}$$

$$\approx \frac{1}{|S|}\sum_{x\in S}\frac{\partial \log p}{\partial \theta_i}\frac{\partial \log p}{\partial \theta_j} \tag{s8}$$

In theory, this could be used to reduce the runtime of smMALA, as the biggest overhead is caused by the exact calculation of the Fisher information matrix. However, in our experiments, it took the MALA only few more steps to converge, the potential speed up would not be sufficient for smMALA to outperform MALA.

S1.3 Positive Definiteness of the Metric Tensor

For a matrix to induce a metric, it must be positive definite. A Fisher information matrix is always positive semi-definite, but we will show that it is in general not positive definite for MHN. In order for eq. (7) to be positive definite, the Hessian of the log-prior $H(\log \mathrm{pr})$ has to be negative definite. As we will show, this does not hold for all priors.

S1.3.1 Non-Positive Definiteness of the FIM for MHN

The Fisher information matrix $\mathcal{I}(\theta)$ is positive semi-definite but in general not positive definite: Vocht (2022) showed that, for a cMHN with $n = 2, 3, 4$, the FIM has a non-trivial nullspace and is therefore not positive definite. The FIM for oMHN is in fact never positive definite: This can be seen by realizing that infinitely many oMHNs have the same probability distribution. There is

a subspace along which the model's parameters can be changed without its probability distribution changing.

Alternatively, one can see that the FIM has a non-trivial nullspace by multiplying it with

$$\begin{pmatrix} 0 & \text{if } i = j \\ 1 & \text{else} \end{pmatrix}_{\substack{i=1,\dots,n+1 \\ j=1,\dots,n}} . \tag{s9}$$

Therefore, it is never positive definite.

This reflects the non-identifiability of an unregularized MHN. Multiple distinct parameter configurations can produce the same probability distribution. This means that the distance induced by the FIM cannot serve as a metric, because it may assign a distance of zero to two points that are not the same.

S1.3.2 (Non-) Negative Definiteness of the Prior

To still use (7) as a metric tensor, this non-identifiability needs to be countered with a log-concave prior. The Hessian $H(\log \mathrm{pr})$ has to be negative definite (This corresponds to the prior pr being strictly log-concave).

This is neither true for the prior corresponding to a L1-penalty eq. (2) nor for the one corresponding to the symsparse penalty eq. (3): The log-Hessians of both have null-rows and columns, giving them a non-trivial nullspace. They are therefore not negative definite.

The log-Hessian of the prior corresponding to the L2-penalty is the identity matrix times some negative scalar and therefore negative definite. The log-Hessian H of the prior corresponding to the symmetrizing L2 penalty (4) is given by

$$H_{\vartheta_{ij}\vartheta_{kl}} = -\lambda \begin{cases} 2 & \text{if } i = k, j = l \\ -1 & \text{if } i = l, j = k, i \neq j \\ 0 & \text{else} \end{cases} \tag{s10}$$

and for any vector x we have

$$x^T H x = -\lambda \Big(\sum_i 2x_{\vartheta_{ii}}^2 + \sum_{i \neq j} (2x_{\vartheta_{ii}}^2 - x_{\vartheta_{ij}} x_{\vartheta_{ji}}) \Big) \tag{s11}$$

$$= -2\lambda \Big(\sum_i x_{\vartheta_{ii}}^2 + \sum_{i<j} (x_{\vartheta_{ii}}^2 - x_{\vartheta_{ij}} x_{\vartheta_{ji}} + x_{\vartheta_{jj}}^2) \Big). \tag{s12}$$

Every summand in the sums is non-negative and 0 if and only if every involved component of x is 0. Therefore H is negative definite.

S2 Pseudocode (sm)MALA

Here we show the pseudocode for the Metropolis-Adjusted Langevin Algorithm (MALA) and for simplified manifold MALA.

Algorithm SA1 MALA for MHN

Input: dataset D, prior π, initial distribution π_{init}, step size ϵ, number of iterations n_{it}

```
samples ← []
draw θ from π_init
samples.append(θ)
for t = 1, ..., n_it do
    θ' ← draw from q(· | θ) = N(θ + ε/2 ∂(log p(θ|D) + log π(θ))/∂θ, εI)
    u ← draw from uniform(0,1)
    if u < p(θ'|D)π(θ')q(θ|θ') / (p(θ|D)π(θ)q(θ'|θ)) then
        θ ← θ'
    end if
    samples.append(θ)
end for
```

Output: samples

Algorithm SA2 smMALA for MHN

Input: dataset D, prior π, initial distribution π_{init}, Fisher information matrix $\mathcal{I}$, Hessian of the log-prior H, step size ϵ, number of iterations n_{it}

```
samples ← []
draw θ from π_init
samples.append(θ)
for t = 1, ..., n_it do
    G(θ) ← I(θ) − H(θ) # metric tensor
    θ' ← draw from q(· | θ) = N(θ + ε/2 G(θ)^{-1} ∂(log p(θ|D) + log π(θ))/∂θ, εG(θ)^{-1})
    u ← draw from uniform(0,1)
    if u < p(θ'|D)π(θ')q(θ|θ') / (p(θ|D)π(θ)q(θ'|θ)) then
        θ ← θ'
    end if
    samples.append(θ)
end for
```

Output: samples

S3 MALA Scaling

In this section, we show how MALA scales with increasing MHN size. We recorded the computation time per MALA step, executed by 10 chains in parallel, for the LUAD dataset, either restricted or extended (by repeating event columns) to increasing numbers of events (fig. SF1). The limit on the number of events depends on the maximum number of active events per individual patient. In practice, the limit is roughly 25 active events on a GPU, while the technical limit is 32.

Note that the number of steps required to reach convergence depends strongly on the specific model.

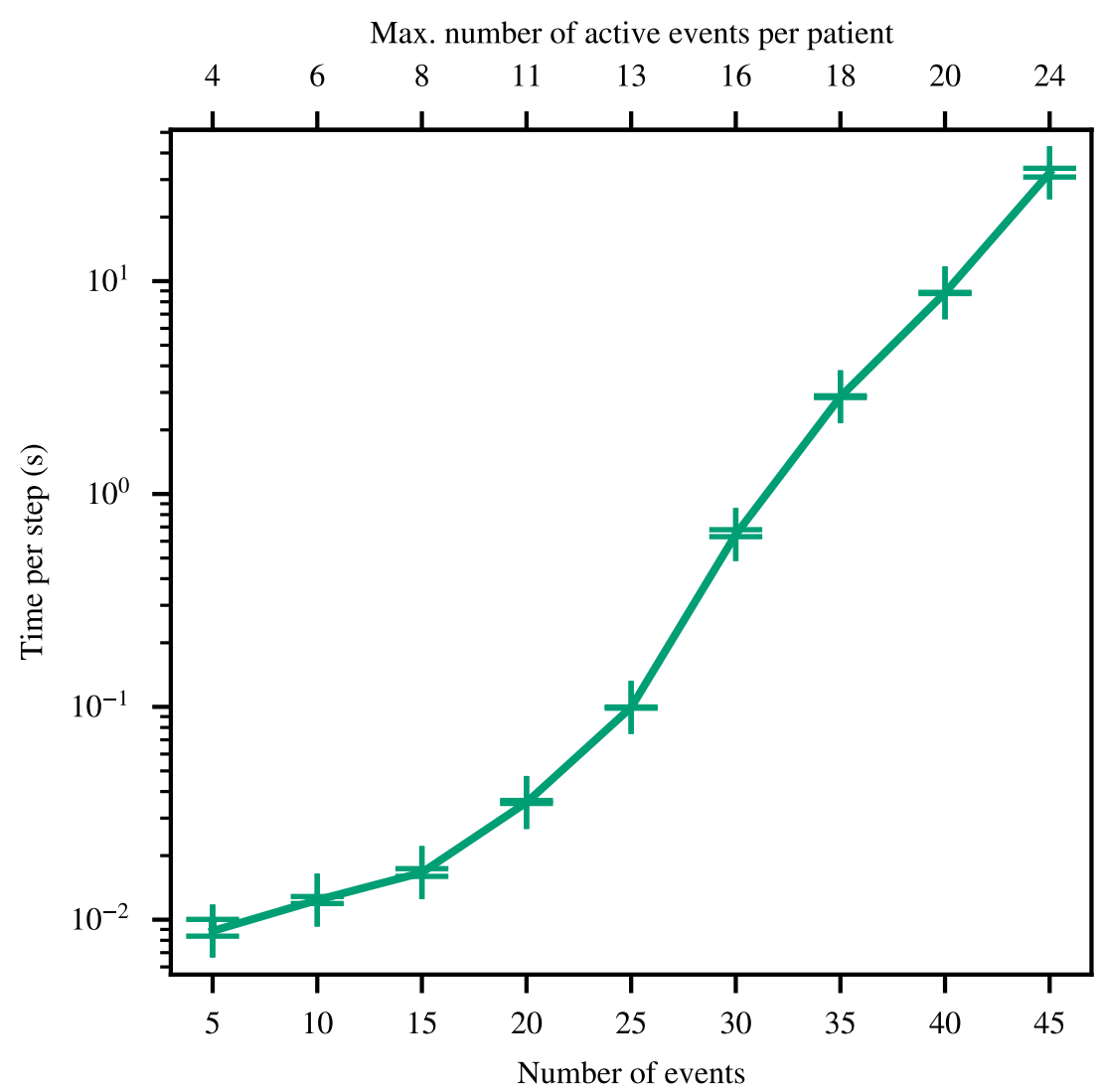

Figure SF1: Mean (minimum and maximum) time for one MALA step, executed by 10 chains in parallel, across 100 repetitions for different event numbers and resulting maximum event numbers per patient. Benchmarks were executed on a Linux computing server equipped with 512 GB RAM and 10 dedicated cores from two AMD EPYC 7453 28-Core Processors as well as an NVIDIA A100 high-performance data center GPU with 80 GB of high-bandwidth HBM2e memory.

S4 Posterior Predictive Checks

In this section, we employ posterior predictive checks Gabry et al. (2019) to assess the fit of the posterior samples.

S4.1 LUAD

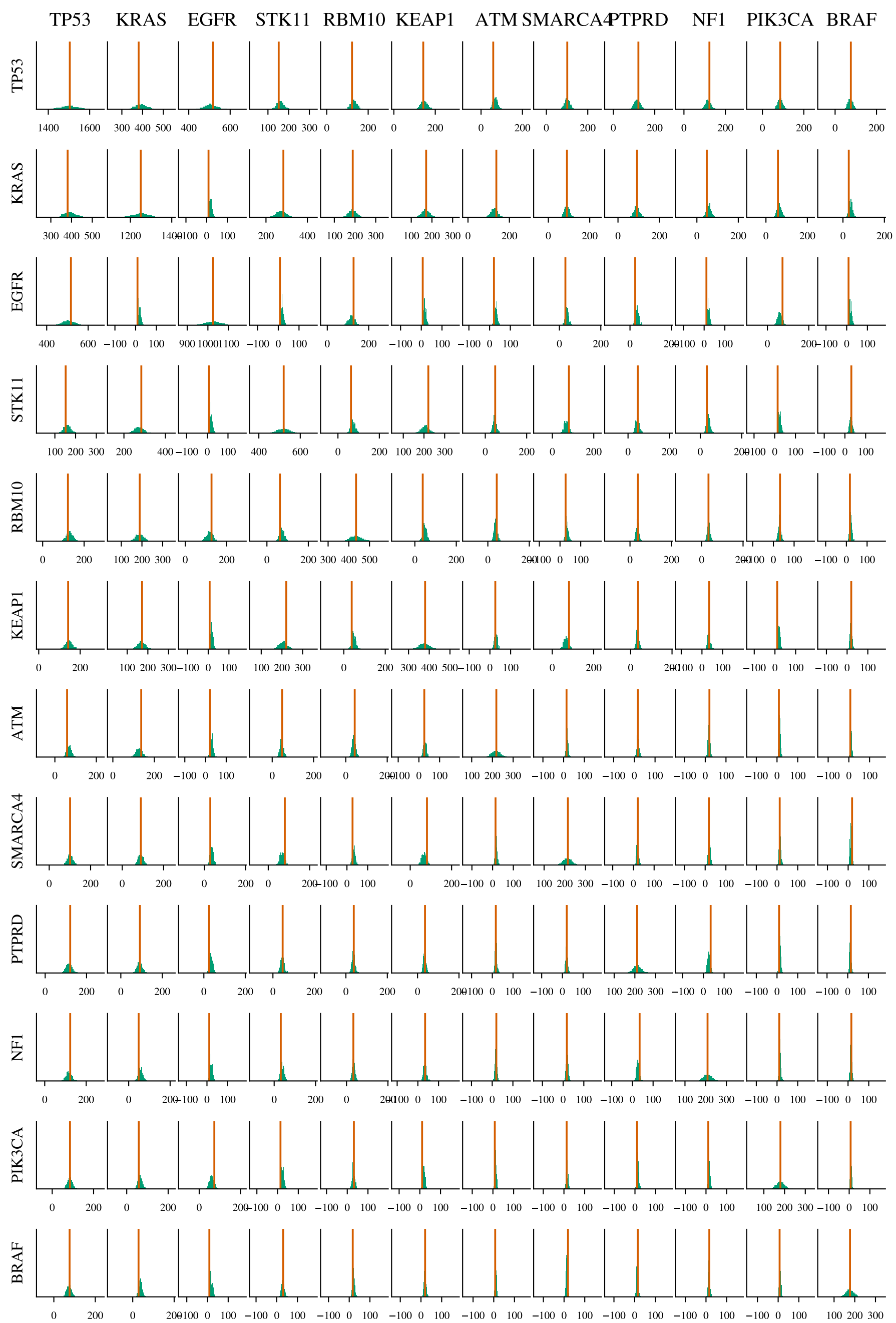

Figure SF2: Histogram cooccurrences from the posterior predictive distribution of the LUAD posterior samples. For every 10th posterior sample, a dataset of the same size as the original dataset was generated. The cooccurrences of the events contribute to the histogram (green). The ground truth cooccurrences from the original dataset are shown in orange.

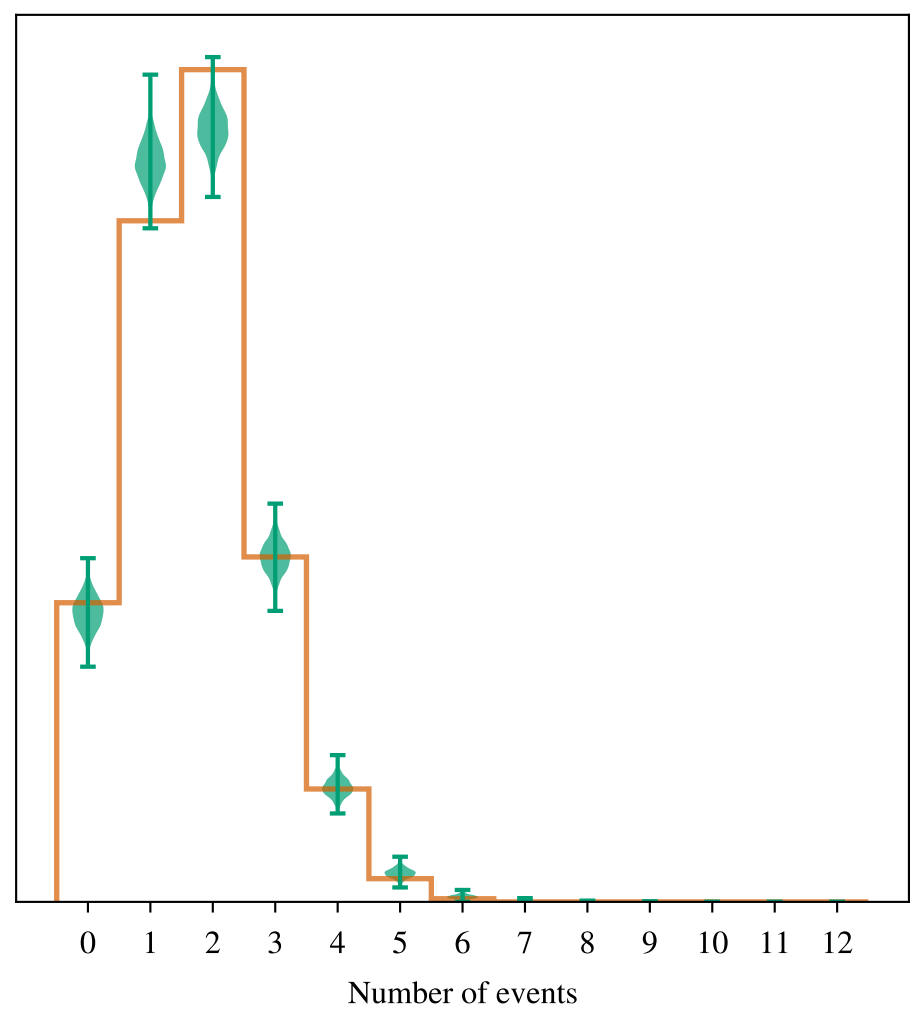

Figure SF3: Event counts from the posterior predictive distribution of the LUAD posterior samples. For every 10th posterior sample, a dataset of the same size as the original dataset was generated. The distribution of their event counts, i. e., number of mutations per sample contribute to the green violin plots. The ground truth distribution of event counts from the original dataset is shown in orange.

S4.2 COAD

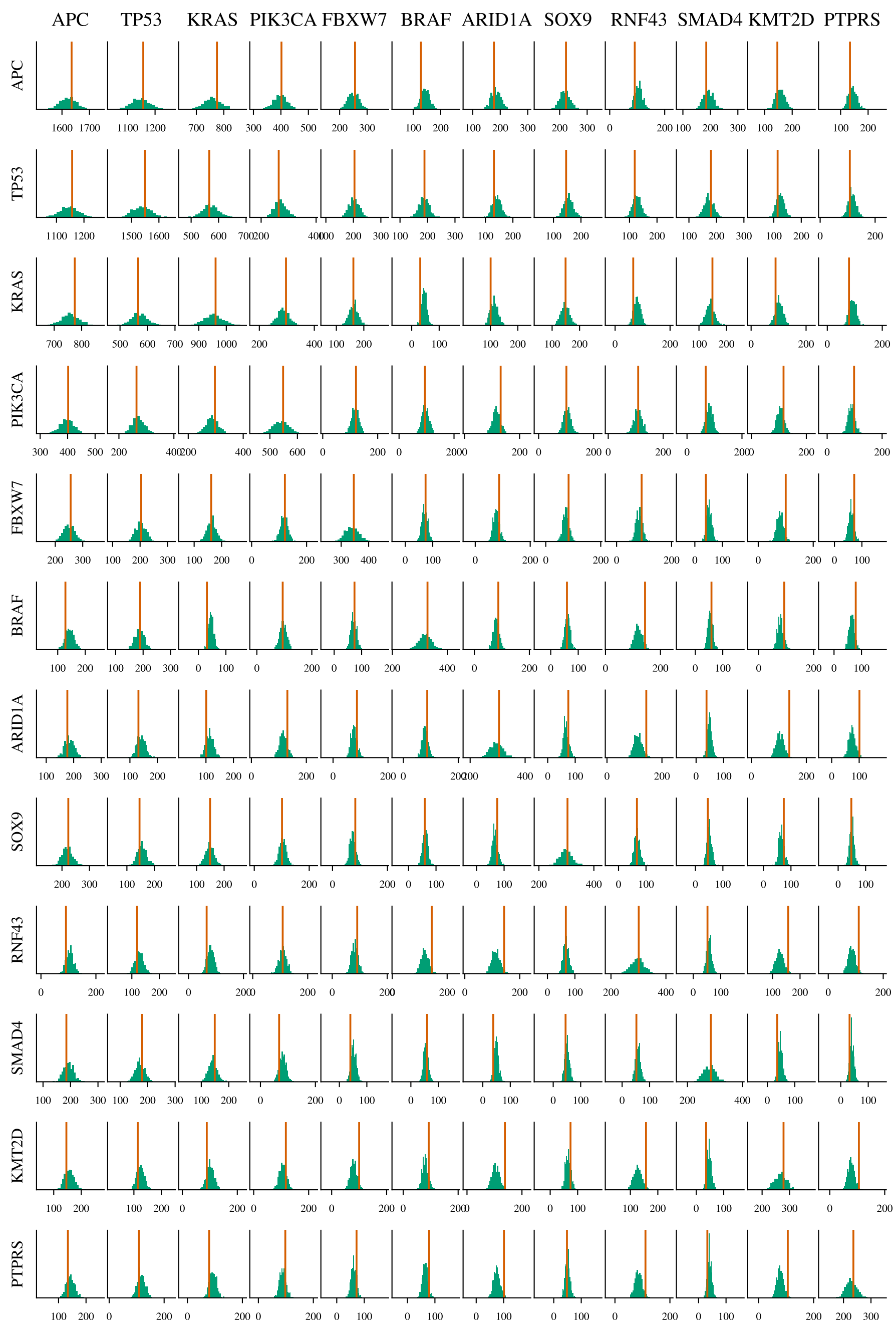

Figure SF4: Histogram cooccurrences from the posterior predictive distribution of the COAD posterior samples. For every 10th posterior sample, a dataset of the same size as the original dataset was generated. The cooccurrences of the events contribute to the histogram (green). The ground truth cooccurrences from the original dataset are shown in orange.

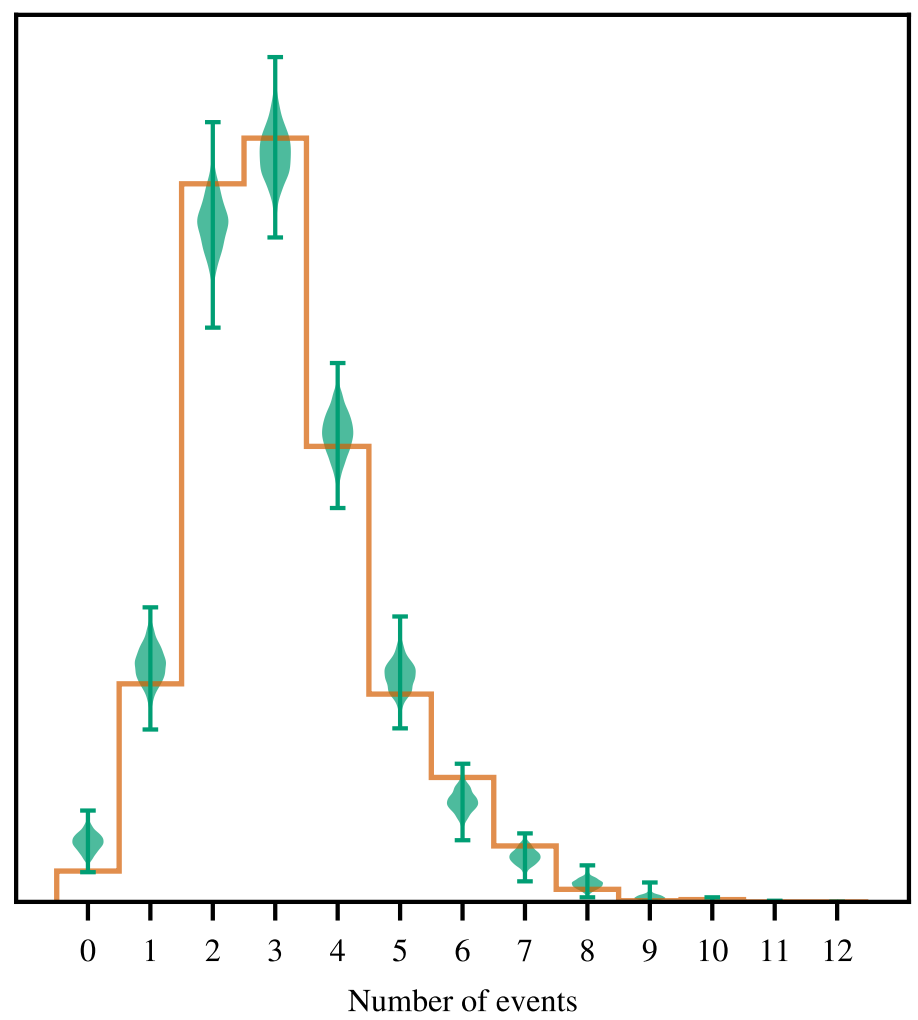

Figure SF5: Event counts from the posterior predictive distribution of the COAD posterior samples. For every 10th posterior sample, a dataset of the same size as the original dataset was generated. The distribution of their event counts, i. e., number of mutations per sample contribute to the green violin plots. The ground truth distribution of event counts from the original dataset is shown in orange.

S5 Mutation Risks

In this section we show the mutation risks for the remaining events as predicted by the MCMC samples.

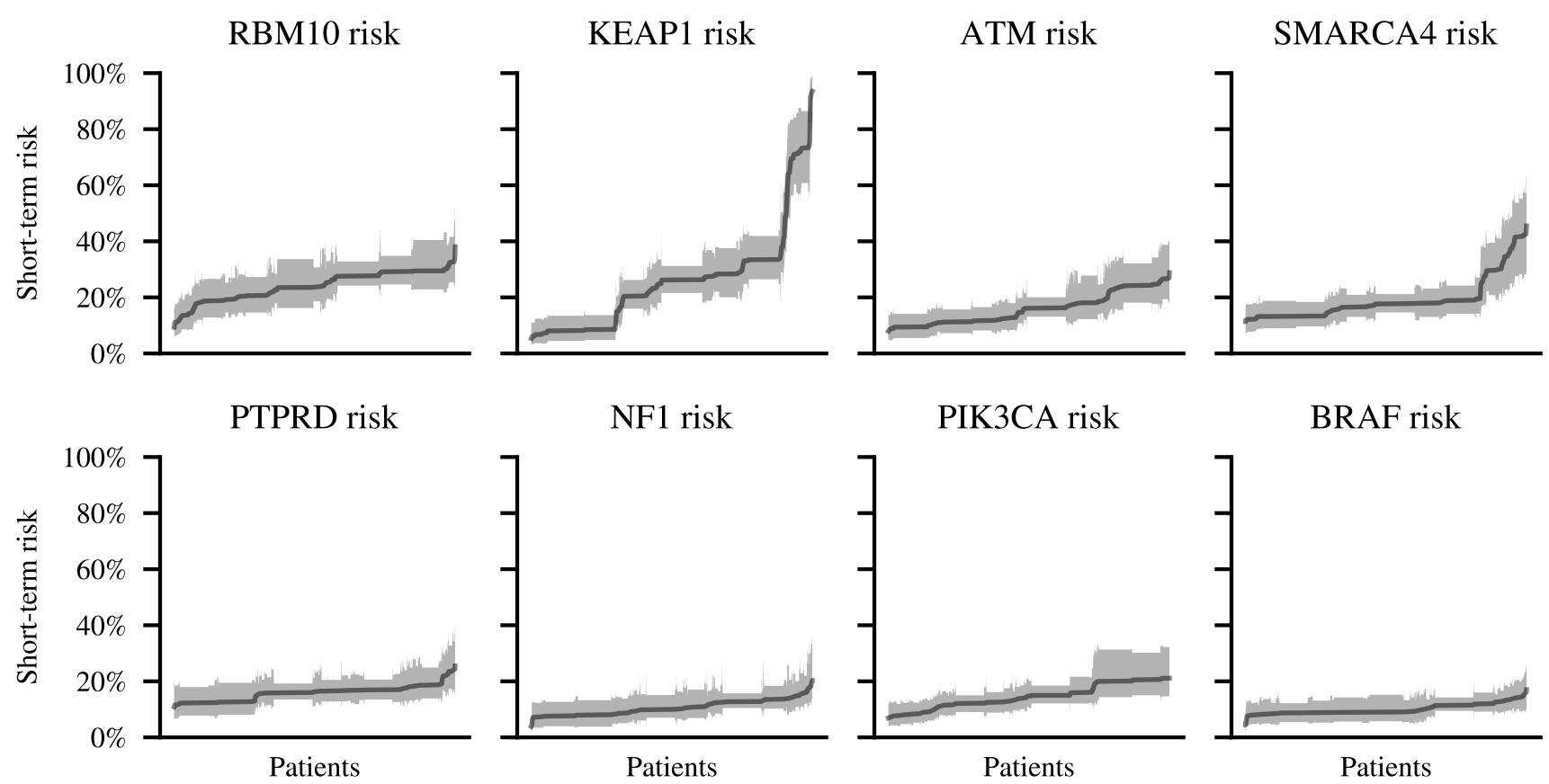

Figure SF6: Predicted short-term mutation risks (median and 90% posterior interval) across patients of the LUAD dataset, predicted by the MCMC samples. For every 100th MCMC sample, 10,000 artificial tumors were sampled for 1 time unit in Markov time to infer a mutation risk. Only patients who do not exhibit this mutation at the time of sequencing are shown and patients are ordered by median risk.

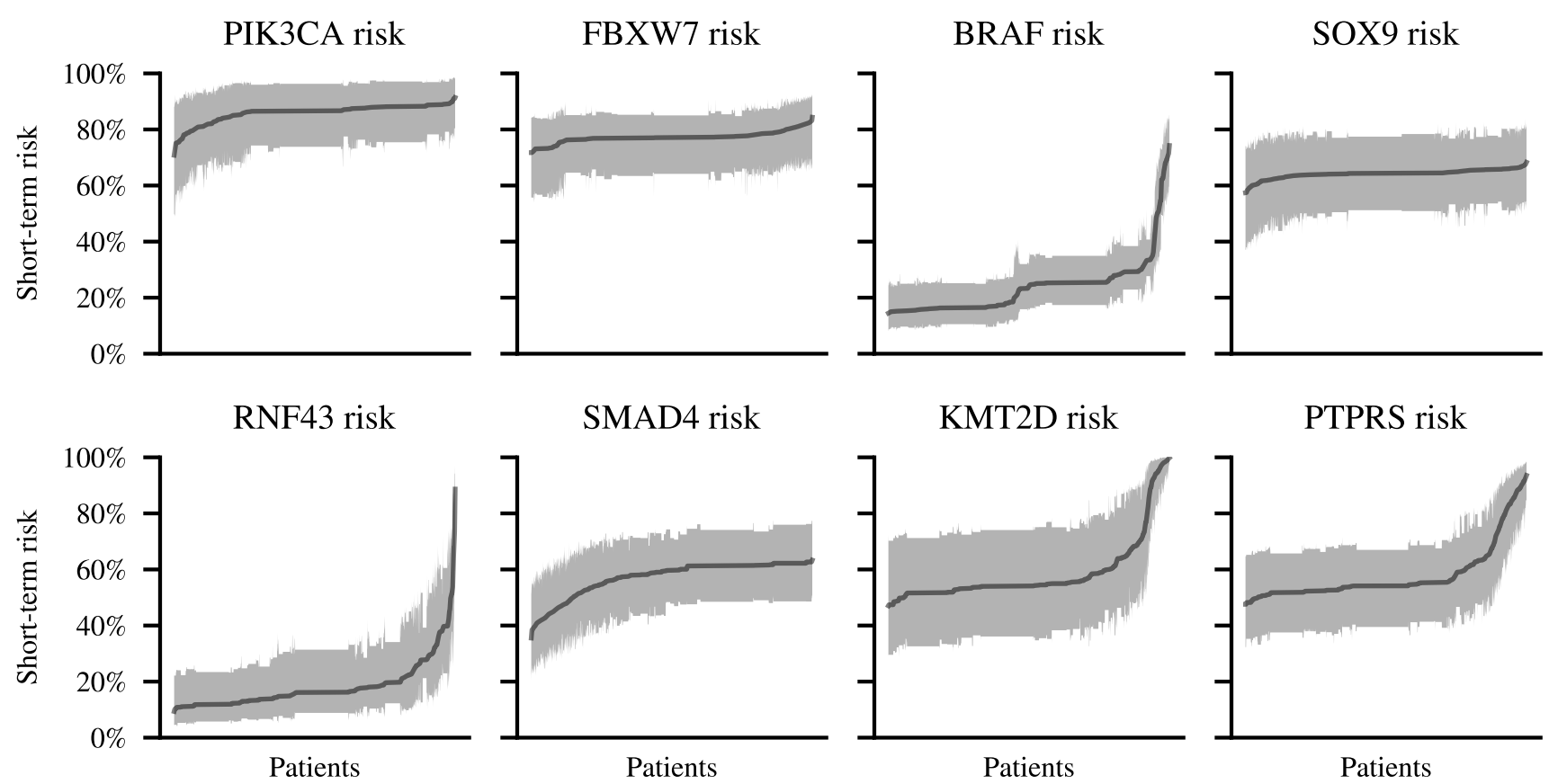

Figure SF7: Predicted short-term mutation risks (median and 90% posterior interval) across patients of the COAD dataset, predicted by the MCMC samples. For every 100th MCMC sample, 10,000 artificial tumors were sampled for 1 time unit in Markov time to infer a mutation risk. Only patients who do not exhibit this mutation at the time of sequencing are shown and patients are ordered by median risk.

S6 Multimodal Posterior Distributions for the L1 MHNs

Here we show the posterior distributions for the LUAD and COAD L1 model, respectively.

S6.1 LUAD

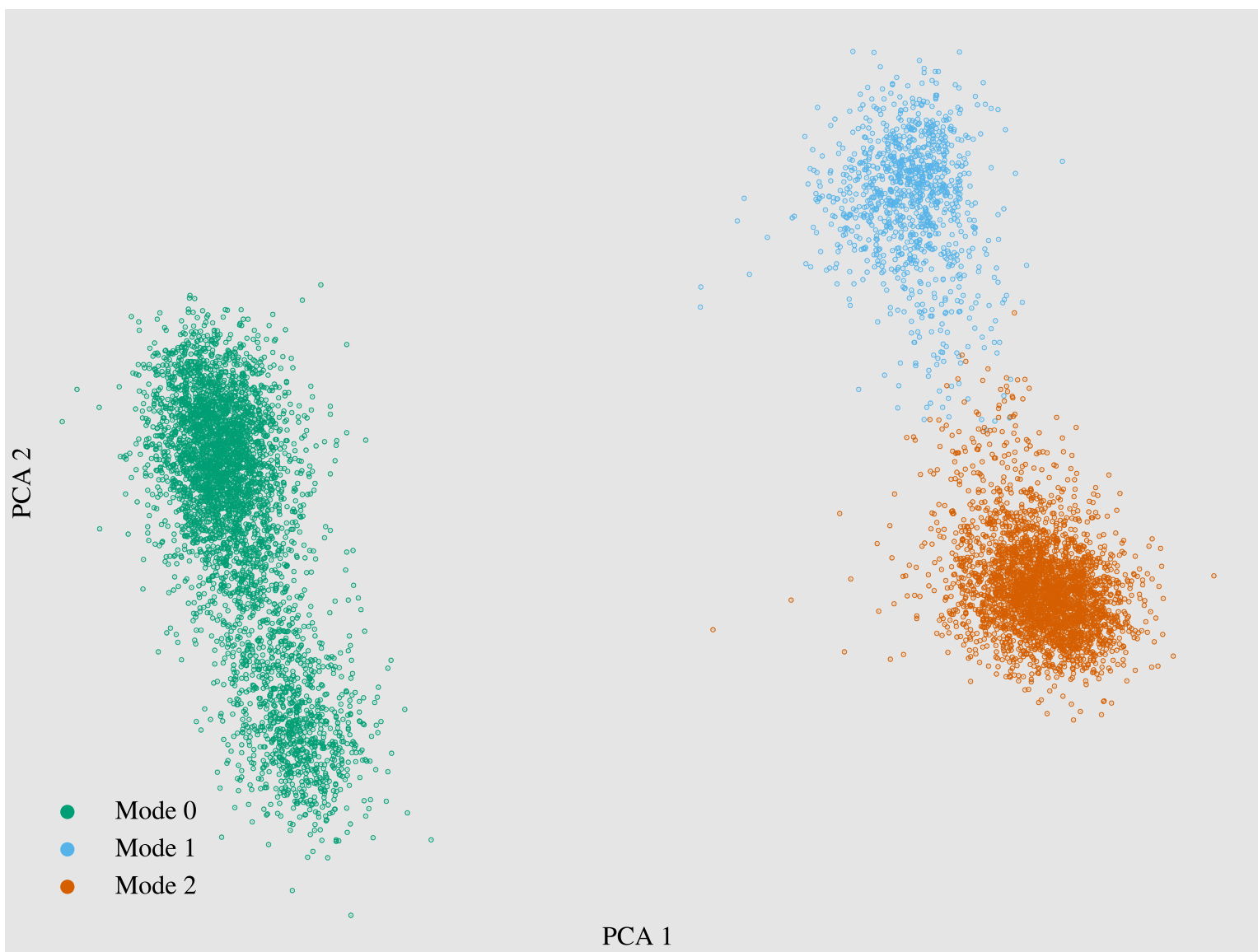

Figure SF8: First two principal components of posterior samples from an MHN trained on the LUAD dataset with a non-symmetrizing L1 penalty. Every 10th sample is shown and colored by cluster. Cluster identities were assigned by applying HDBSCAN to a 2-dimensional UMAPping.

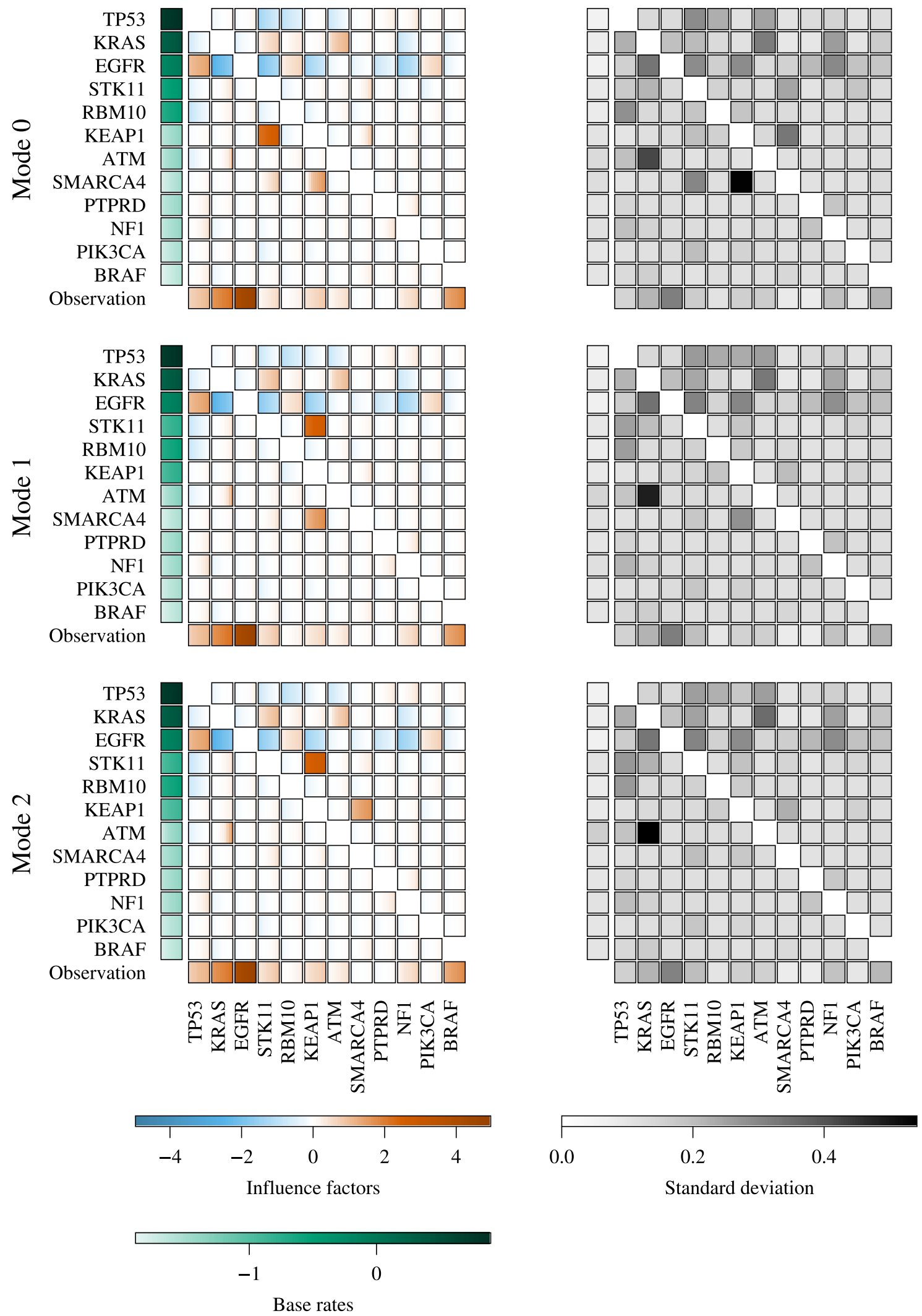

Figure SF9: Distribution and standard deviation of the parameter matrix for the modes of the posterior samples of an MHN trained on the LUAD dataset with the L1 penalty (fig. SF8). Each cell in the left plots shows the 90% posterior interval of the corresponding parameter as a color gradient. E. g., the leftmost color in a cell corresponds to the 5% quantile of the parameter's distribution, the middle color to its 50% quantile, the rightmost color to its 95% quantile. Influence factors and observation rates are shown in blue/orange and base rates are shown in green. Each cell in the right plots shows the standard deviation of the corresponding parameter. Note that for better visibility in the parameter matrices we are using different color scales than in main article.

S6.2 COAD

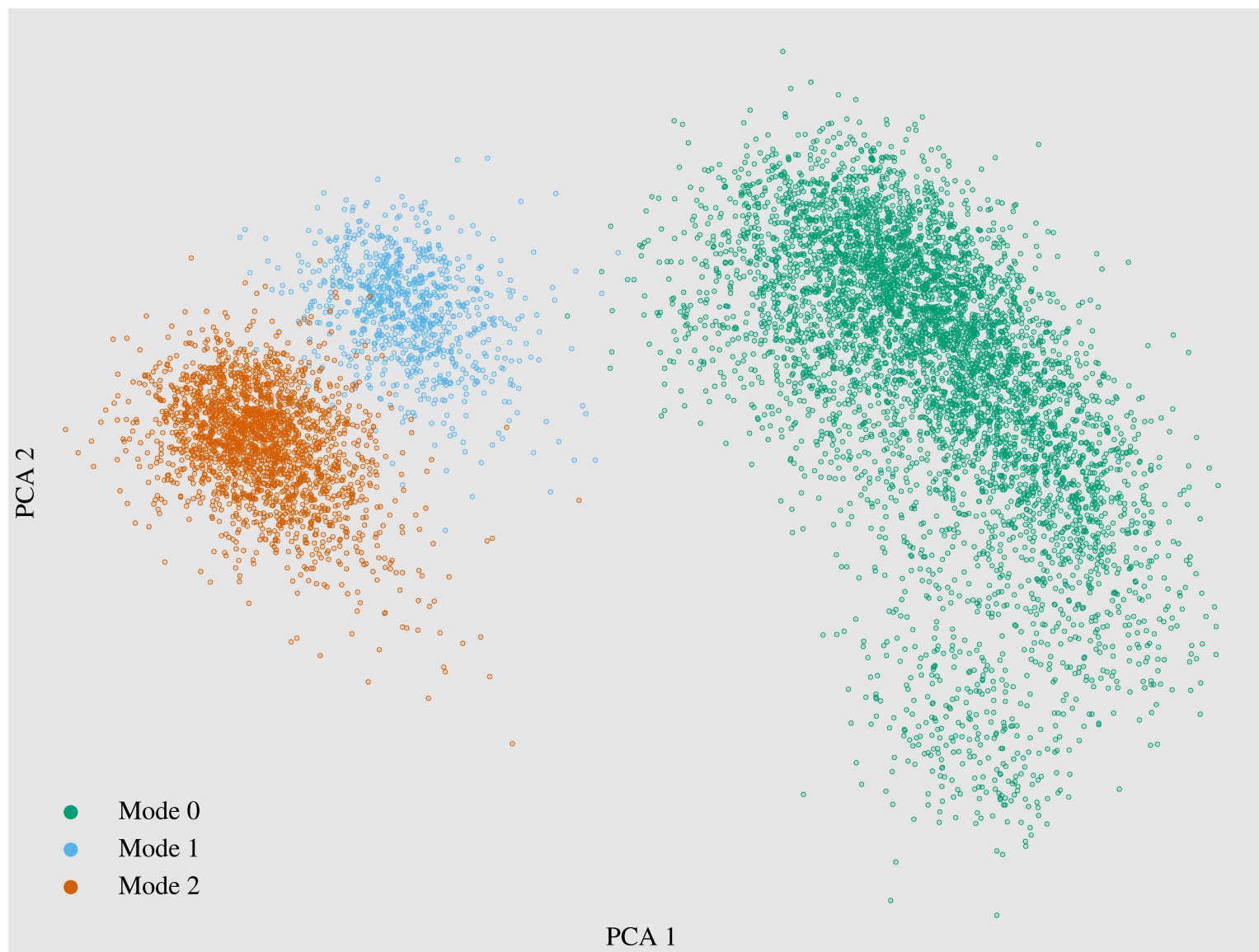

Figure SF10: First two principal components of posterior samples from an MHN trained on the COAD dataset with a non-symmetrizing L1 penalty. Every 10th sample is shown and colored by cluster. Cluster identities were assigned by applying HDBSCAN to a 2-dimensional UMAPping.

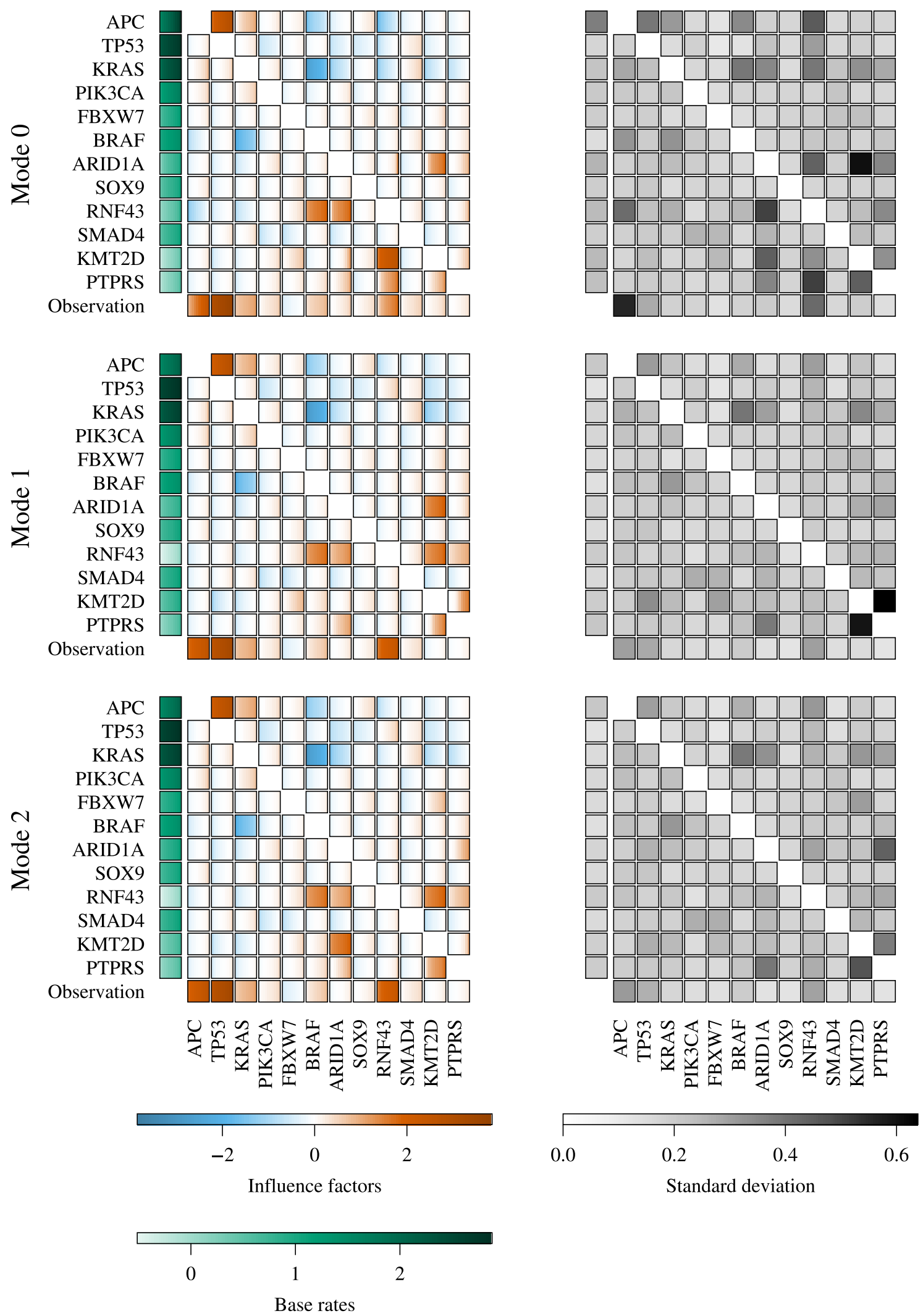

Figure SF11: Distribution and standard deviation of the parameter matrix for the modes of the posterior samples of an MHN trained on the COAD dataset with the L1 penalty (fig. SF10). Each cell in the left plots shows the 90% posterior interval of the corresponding parameter as a color gradient. E. g., the leftmost color in a cell corresponds to the 5% quantile of the parameter's distribution, the middle color to its 50% quantile, the rightmost color to its 95% quantile. Influence factors and observation rates are shown in blue/orange and base rates are shown in green. Each cell in the right plots shows the standard deviation of the corresponding parameter. Note that for better visibility in the parameter matrices we are using different color scales than in main article.

References

J. Gabry, D. Simpson, A. Vehtari, M. Betancourt, and A. Gelman. Visualization in bayesian workflow. *Journal of the Royal Statistical Society Series A: Statistics in Society*, 182(2):389–402, Jan. 2019. ISSN 1467-985X. doi: 10.1111/rssa.12378.

M. Girolami and B. Calderhead. Riemann manifold langevin and hamiltonian monte carlo methods. *Journal of the Royal Statistical Society Series B: Statistical Methodology*, 73(2):123–214, 03 2011. ISSN 1369-7412. doi: 10.1111/j.1467-9868.2010.00765.x.

S. Vocht. Identifiability of Mutual Hazard Networks, 2022. Bachelor thesis.
